# Magnetic Agarose Microspheres/Hyaluronic Acid Hydrogel as a Trackable Bulking Agent for Vesicoureteral Reflux Treatment

**DOI:** 10.3389/fbioe.2021.746609

**Published:** 2021-10-07

**Authors:** Hong Chen, Pan Wu, Hong Xu, Changchun Wang

**Affiliations:** ^1^ Children’s Hospital of Fudan University, Shanghai, China; ^2^ State Key Laboratory of Molecular Engineering of Polymers, Department of Macromolecular Science, and Laboratory of Advanced Materials, Fudan University, Shanghai, China

**Keywords:** bulking agent, magnetic microspheres, MRI contrast agent, biosecurity, vesicoureteral reflux, subureteral transurethral injection

## Abstract

Vesicoureteral reflux (VUR) is one of the most common congenital anomalies in the kidney and the urinary tract. Endoscopic subureteral injection of a bulking agent has become popular in VUR treatment due to its high success rates, few complications, and a straightforward procedure. In this study, a novel magnetic bulking agent was prepared by embedding Fe_3_O_4_ magnetic nanoparticles in cross-linked agarose microspheres with diameters of 80–250 μm and dispersing the magnetic microspheres in a hyaluronic acid hydrogel. The bulking agent has good biocompatibility and biosecurity validated by the tests of cytotoxicity, *in vitro* genotoxicity, animal irritation, skin sensitization, acute systemic toxicity, and pathological analysis after the injection of the bulking agent extract solution into healthy mice as well as injection of the bulking agent into VUR rabbits. The VUR rabbits were created by incising the roof of the intravesical ureter to enlarge the ureteral orifice. The success rate of the bulking agent in treating VUR rabbits using a subureteral transurethral injection technique was 67% (4/6) or 80% (4/5, excluding the unfinished rabbit), and no migrated particles were found in the organs of the rabbits. The transverse relaxation rate of the bulking agent was 104 mM^−1^s^−1^. After injection, the bulking agent was long-term trackable through magnetic resonance imaging that can help clinicians to inspect the VUR treatment effect. For the first time, this study demonstrates that the bulking agent with a long-term stable tracer is promising for endoscopic VUR treatment.

## Introduction

Vesicoureteral reflux (VUR) is one of the most common congenital anomalies in the kidney and the urinary tract that occurs in 0.4–1.8% of all children (Skoog et al., 2010; Renkema et al., 2011). VUR is associated with an increased risk of urinary tract infection (Arlen and Cooper, 2015). Children with persistent VUR are predisposed to long-term sequelae, including hypertension, preeclampsia, proteinuria, chronic renal insufficiency, and even end-stage renal disease (Simoes et al., 2007; Montini et al., 2011). The aim of the treatment of a child with VUR is to prevent recurrent febrile urinary tract infection and new renal damage, and to minimize the morbidity of the therapy and follow-up procedures (Peters et al., 2010). Surgical intervention is often the fully effective approach in VUR treatment. Among surgical interventions, endoscopic subureteral injection of a bulking agent has the advantages of the least invasion and shortest hospital stay. The endoscopic treatment of VUR is to create a solid support behind the intravesical ureter using the injected bulking agent, therefore to elongate the intramural tunnel of the ureter and support the ureteral orifice (Johnston et al., 2016; Blais et al., 2017). The endoscopic injection of the bulking agent has become increasingly popular in VUR treatment due to its high success rates, few complications, and a straightforward procedure (Kirsch et al., 2006; Elmore et al., 2008; Molitierno et al., 2008; Herbst et al., 2014; Blais et al., 2017). For the endoscopic treatment of VUR, the bulking agent is a key to success (Keshel et al., 2020). An ideal bulking agent should be able to pass through the syringe needle and have long-term structural stability, biosecurity, and biocompatibility (Kershen and Atala, 1999; Kirsch and Arlen, 2014; Moore and Bolduc, 2014; Kim et al., 2018). Several bulking agents have been used in the VUR treatment, in which dextranomer/hyaluronic acid (Dx/HA, Deflux^®^) is the most widely used one (Cerwinka et al., 2008; Moore and Bolduc, 2014; Johnston et al., 2016; Ceylan et al., 2021). However, the biodegradability of Dx/HA results in a relatively higher long-term VUR recurrence rate (Lee et al., 2009; Alizadeh et al., 2019). The nondegradable bulking agent polyacrylate polyalcohol copolymer (PPC) presents better overall success rate than Dx/HA (Alizadeh et al., 2019), but severe fibrosis at the injection site and an obstructive complication occur at a relatively higher rate (Kim et al., 2017; Sizonov et al., 2020).

In this study, for the first time, we introduced magnetic nanoparticles as a long-term stable tracer in the bulking agent for endoscopic VUR treatment as well as for the inspection of the treatment effect using magnetic resonance imaging (MRI). A novel magnetic bulking agent was prepared by dispersing cross-linked magnetic agarose (Agar) microspheres in the hyaluronic acid (HA) hydrogel. The magnetic agarose microspheres were produced by embedding magnetic Fe_3_O_4_ nanoparticles in the microspheres. The bulking agent was named Fe_3_O_4_@Agar/HA. As the function in the commercial Dx/HA bulking agent (Diamond and Mattoo, 2012; Kim et al., 2017), the hyaluronic acid hydrogel was the carrier of the magnetic agarose microspheres. Agarose, extracted from red algae, is mainly eliminated by the macrophages in the body due to a lack of the degrading enzyme (Luo and Tang, 2015; Park et al., 2020). The magnetic agarose microspheres we prepared were cross-linked and had diameters of 80–250 μm to prevent the microspheres from dissociation, elimination by macrophages, and diffusion through macrophage migration (Molitierno et al., 2008). Fe_3_O_4_ nanoparticles have good biocompatibility and biosecurity, and are used as MRI contrast agents (Laurent et al., 2008). The cross-linked agarose microspheres can protect the embedded Fe_3_O_4_ nanoparticles from decomposition and migration that enable the bulking agent to have a long-term MRI contrast signal for tracking. The biocompatibility and biosecurity of the bulking agent Fe_3_O_4_@Agar/HA were assessed by cytotoxicity, *in vitro* genotoxicity, animal irritation, skin sensitization, acute systemic toxicity, and pathological analysis, in accordance with the Chinese National Standard of Biological Evaluation of Medical Devices GB/T 16886.1-2011/ISO 10993-1:2009 (AQSIQ and SAC, 2011a). The VUR rabbits were created by incising the roof of the intravesical ureter to enlarge the ureteral orifice. The effect in treating VUR rabbits and MRI in tracking the injected bulking agent in the rabbits were evaluated to validate that the bulking agent is a promising one for synchronous VUR treatment and inspection.

## Material and Methods

### Materials

Agarose (Agar, AR, BIOWEST AGAROSE) was purchased from Gene Company Limited (Shanghai, China). Hyaluronic acid (HA, 97%) was from Sa En Chemical Technology (Shanghai) Co., Ltd. (Shanghai, China). The other chemicals with analytical grade were from Sinopharm Chemical Reagent Co. Ltd. (Shanghai, China).

### Preparation and Characterization of the Bulking Agent

#### Preparation of Cross-linked Agarose Microspheres Embedded With Fe_3_O_4_ Nanoparticles (Fe_3_O_4_@Agar)

Fe_3_O_4_ magnetic nanoparticles were prepared by a coprecipitation method (Xu et al., 2004; Xu et al., 2007). The obtained nanoparticles were collected through a magnetic field and washed repeatedly with deionized water. The Fe_3_O_4_ magnetic nanoparticles were observed on a transmission electron microscope (T20, FEI, United States).

Fe_3_O_4_@Agar microspheres were produced by an inverse suspension method. Typically, 0.24 g Fe_3_O_4_ (wet weight), 0.25 g agarose powder, and 5.76 g deionized water were added in a 25-ml flask. The mixture was sonicated (JP-010T, SKYMEN, China) for 5 min and heated to 95°C, then 0.5 ml of 2 M NaOH was added. The resultant suspension was mechanically stirred at 250 rpm for 20 min to dissolve the agarose. The emulsifier span-80 of 0.5 g was added in 25 ml n-octane. The n-octane solution was mechanically stirred at 1,000 rpm and meanwhile heated to 65°C, and then the Fe_3_O_4_ and the agarose mixed aqueous suspension was transferred into the n-octane solution. After stirring at 1,000 rpm and 65°C for 0.5 h, the suspension was cooled to 55°C, and 0.5 g of the cross-linking agent epichlorohydrin was added. The cross-linking reaction proceeded at a stir speed of 300 rpm for 1 h. After the reaction, the suspension was stationary at room temperature. The cross-linked magnetic agarose microspheres in the lower layer were collected and washed with 20 v% ethanol–water solution several times, filtered through a sieving sieve, and washed with deionized water successively to obtain purified Fe_3_O_4_@Agar microspheres with diameters of 80–250 µm. The morphology and size of the purified and undried microspheres were observed on a microscope (BX51, Olympus, Japan).

#### Preparation of Fe_3_O_4_@Agar/HA Magnetic Hydrogel

The purified and undried Fe_3_O_4_@Agar microspheres were added in deionized water with the concentration equivalent to 50 mg/ml dried microspheres, and then hyaluronic acid powder was added with the concentration of 15 mg/ml. The mixture was mechanically stirred overnight to obtain a uniform Fe_3_O_4_@Agar/HA magnetic hydrogel. The magnetic hydrogel was sterilized by autoclaving and dispensed into a 1-ml or 2-ml syringe before use.

#### Magnetic Resonance Imaging Characterization of Fe_3_O_4_@Agar/HA

The Fe_3_O_4_@Agar/HA samples with the final Fe concentrations of 0, 0.0008, 0.0016, 0.0032, 0.0064, 0.0128, 0.0256, and 0.0512 mM were, respectively, prepared by adding 0, 0.0075, 0.015, 0.03, 0.06, 0.12, 0.24, and 0.48 g of the Fe_3_O_4_ nanoparticles in the Fe_3_O_4_@Agar microspheres, while the agarose and hyaluronic acid concentrations were unchanged. The transverse relaxation time (T_2_)-weighted magnetic resonance signal intensities of the Fe_3_O_4_@Agar/HA samples were acquired on an MRI instrument (BioSpec 70/20 USR, Bruker, Germany). The parameters were repeating time 3,000 ms, echo time 105.4 ms, and field of view 4.00 cm. The transverse relaxation rate of Fe_3_O_4_@Agar/HA was obtained by a linear fitting of 1/T_2_ *versus* Fe concentration.

### Biological Evaluations of the Bulking Agent

#### Cytotoxicity

Cytotoxicity was evaluated using the MTT method (AQSIQ and SAC, 2017a). The cell line was mouse fibroblast L929 from the Cell Bank of Chinese Academy of Science (Shanghai, China). The Fe_3_O_4_@Agar/HA extract solution was prepared by immersing 0.2 g Fe_3_O_4_@Agar/HA in a 1 ml MEM medium containing 10% fetal bovine serum (Fisher Scientific International Inc., Logan, UT, United States) at 37°C and 5% CO_2_ for 24 h, and the solution was immediately used after filtration. The positive control was a polyurethane film containing 0.1% zinc diethyl dithiocarbamate, and the negative control was a high-density polyethylene film (AQSIQ and SAC, 2017a). Their extract solutions were prepared as the solution of Fe_3_O_4_@Agar/HA. The cells were incubated with one of the extract solutions for 24 h to evaluate the cytotoxicity. Six parallel experiments were performed for each condition.

#### *In Vitro* Genotoxicity

*Salmonella typhimurium* histidine-deficient strains TA98, TA100, TA102, TA1535, and TA1537 were obtained from MOLTOX^®^ Molecular Toxicology, Inc. (Boone, NC, United States). The extract solution was prepared by immersing 0.2 g Fe_3_O_4_@Agar/HA in 1 ml saline and incubating the solution at 50°C for 72 h with shaking. The extract solution was used without filtration within 6 h. The metabolic activation, rat liver homogenate (S9), was from CHI Scientific (Boston, MA, United States). The genotoxicity test was performed using the bacterial reverse mutation test (Ames test) by a plate incorporation method (Hamada et al., 1994; Zeiger, 2019). Three parallel experiments were performed for each condition.

#### Animal Irritation

The animal experiments of this study were reviewed and approved by the Ethics Committee of Children’s Hospital, Fudan University [ethical approval number: (2018) 119]. All the animals were housed in full compliance with the Chinese National Standard GB14925-2010 (AQSIQ and SAC, 2010) and acclimated to the environment for more than 5 days before the experiment. The animals had free access to standard diet and water.

Three healthy New Zealand young adult male rabbits from the Songlian Laboratory Animal Farm (Shanghai, China) were used for the single exposure test. The fur of the both sides of the spine (approximately 10 cm × 15 cm) was clipped at 24 h before the test. For each rabbit, the two right sites were covered with 0.5 ml Fe_3_O_4_@Agar/HA, and the two left sides were blank control. Each of the application sites was covered with 2.5 cm × 2.5 cm sterile gauze and wrapped with a bandage for 4 h. After that the bulking agent was removed by washing with saline, and the sites were carefully wiped dry. The appearance of each application site was recorded at 1, 24, 48, and 72 h after the removal of Fe_3_O_4_@Agar/HA. The primary irritation index of Fe_3_O_4_@Agar/HA was calculated as described in GB/T 16886.10-2017/ISO 10993-10:2010 (AQSIQ and SAC, 2017b).

#### Skin Sensitization

Healthy Harley guinea pigs in their early adulthood (260–430 g) were obtained from the Laboratory Animal Center of Southern Medical University (Guangzhou, Guangdong, China). The Fe_3_O_4_@Agar/HA extract solution was prepared as described in the *in vitro* genotoxicity section, and used without filtration within 24 h. Freund’s complete adjuvant was from STC (Dongguan) Company Limited (Dongguan, Guangdong, China). Five samples were used in the guinea pig maximization test (AQSIQ and SAC, 2017b). Sample A was the Freund’s complete emulsion prepared by mixing the adjuvant with saline at a 1:1 volume ratio. Sample B was the extract solution without dilution. Sample C was the mixed solution of sample A and sample B with a 1:1 volume ratio. Sample D was saline. Sample E was the mixed solution of sample A and saline with a 1:1 volume ratio. Ten guinea pigs were treated with the samples A, B, and C (two sites for each sample and six sites for each guinea pig), and five guinea pigs were treated with samples A, D, and E as the control. The test procedure including an intradermal induction phase, a topical induction phase, and a challenge phase was the same as described in GB/T 16886.10-2017/ISO 10993-10:2010 (AQSIQ and SAC, 2017b). At 24 and 48 h after the removal of the dressings, the erythema and edema of the challenge skin sites were observed and graded according to the Magnusson and Kligman grading (AQSIQ and SAC, 2017b).

#### Acute Systemic Toxicity

Healthy young adult KM mice (nulliparous female) were purchased from the Laboratory Animal Center of Southern Medical University. The mice were divided into two groups with five in each. The Fe_3_O_4_@Agar/HA extract solution was prepared as described in the *in vitro* genotoxicity section, and used without filtration within 24 h. In the extract solution group, the extract solution was injected *via* the caudal vein at a single dosage of 50 ml/kg. In the control group, the same volume saline was injected. Immediately after the injection and at 4, 24, 48, and 72 h postinjection, death or systemic toxicity was observed (AQSIQ and SAC, 2011b). The mice were weighed before as well as 1, 2, and 3 days after the injection. After that, the mice were sacrificed, the gross pathological changes were observed, and the organs such as the heart, liver, spleen, lung, and brain were taken out for pathological analysis by paraffin section and microscopic examination methods.

### Treatment of Vesicoureteral Reflux Rabbits With the Bulking Agent

#### Vesicoureteral Reflux Model Rabbits

Healthy New Zealand male rabbits (8–10 weeks, 2.2–2.6 kg) were acclimated to the environment for more than 1 week. The rabbit was anesthetized with an intramuscular injection of 25 mg/kg ketamine (Jiangsu Hengrui Medicine Co., Ltd., Lianyungang, Jiangsu, China), and then the roof of the left intravesical ureter was incised to create VUR (Baek et al., 2010; Mangera and Edhem, 2012). Four weeks after the surgery, voiding cystourethrogram (VCUG) was acquired on an X-Ray System (AXIOM Iconos R200, SIEMENS, Germany) to confirm the reflux (Sencan et al., 2008).

#### Subureteral Transurethral Injection in Vesicoureteral Reflux Rabbits

A total of 14 VUR rabbits (Grade II-III) were randomly divided into three groups to receive the following operations: 1) six rabbits in the bulking agent injection group were injected with Fe_3_O_4_@Agar/HA; 2) four rabbits in the saline control group were injected with saline; and 3) four rabbits in the sham-operation control group were anesthetized, and their bladders were opened but no injections were performed. After being anesthetized with 25 mg/kg ketamine by intramuscular injection, the bladder of the VUR rabbit was opened through the original surgical incision and the trigone was exposed. Then, 0.2–0.3 ml of the bulking agent or saline was immediately injected through a 0.45-mm ID needle and 1 ml syringe into the submucosal plane beneath the left ureteral orifice at the 6 o’clock position (O’donnell and Puri, 1984). A dosage of 50 mg/kg/day ceftriaxone (Shanghai Roche Pharmaceuticals Ltd., Shanghai, China) was given intramuscularly at 30 min before the operation as well as on the first and second days after the operation as a prophylactic antibiotic therapy.

#### Post Vesicoureteral Reflux Treatment Evaluation

After 4 weeks of the operation, the following investigations were performed in succession for the rabbits: 1) VCUG examination to evaluate the therapeutic effect; 2) T_2_-weighted MRI (Siemens Prisma 3.0T, Germany) to observe the injected bulking agent; the rabbits being anaesthetized by intraperitoneal injection with 10% chloral hydrate at a dosage of 3.5 ml/kg and examined at the conditions of field strength 3.00 T, slice thickness 2 mm, repetition time 6,000 ms, and echo time 108.0 ms; 3) pathological analysis of the paraffin sections of the organs to evaluate the biocompatibility and migration of the bulking agent; three samples being randomly acquired from each organ of the liver, spleen, gallbladder, pancreas, heart, brain, bladder, and bilateral kidneys, ureteropelvic junctions, ureterovesical junctions, and middle ureters.

### Statistical Analysis

The research data were analyzed using the Statistical Package for the Social Sciences software (SPSS, version 18, SPSS Inc., Chicago, IL, United States). An independent sample t-test or Fisher’s exact test was used for comparison between groups. *p* values less than 0.05 were considered statistically significant.

## Results

### Preparation and Characterization of Magnetic Bulking Agent Fe_3_O_4_@Agar/HA

**FIGURE 1:**
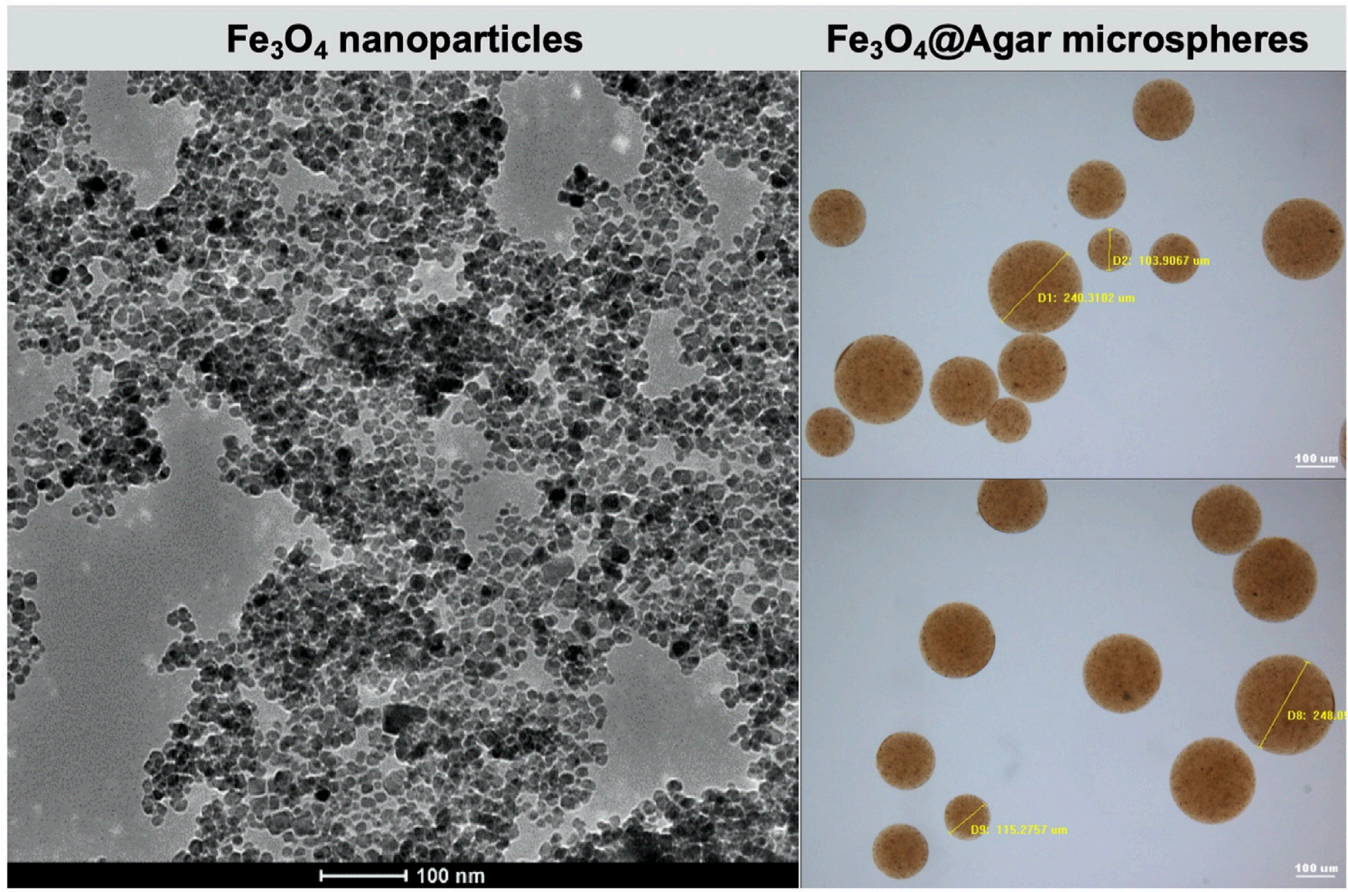
Transmission electron microscopy image of the Fe_3_O_4_ nanoparticles and optical microscopy images of the Fe_3_O_4_@Agar microspheres.

Fe_3_O_4_@Agar/HA was prepared by embedding Fe_3_O_4_ magnetic nanoparticles into cross-linked agarose microspheres Fe_3_O_4_@Agar and dispersing Fe_3_O_4_@Agar in a hyaluronic acid hydrogel. As shown in Figure 1, the prepared Fe_3_O_4_ magnetic nanoparticles had the sizes of 10–20 nm. The diameters of the magnetic agarose microspheres Fe_3_O_4_@Agar were 80–250 μm, which were the same as the diameters of dextranomer microspheres in the commercial bulking agent Dx/HA (Kim et al., 2017). There was no obvious change in terms of the diameter and morphology of the Fe_3_O_4_@Agar microspheres before and after the sterilization, as shown in Supplementary Figure S1, confirming that the magnetic agarose microspheres were stable undergoing sterilization by autoclaving. The concentrations of Fe_3_O_4_@Agar microspheres and hyaluronic acid in Fe_3_O_4_@Agar/HA were also the same as the concentrations of dextranomer microspheres and hyaluronic acid in Dx/HA. For Dx/HA, a localized mound is created after the submucosal injection, and hyaluronic acid undergoes gradual absorption and is replaced by a collagen matrix, forming a persistent tissue implant at the injection site (Diamond and Mattoo, 2012; Kim et al., 2017). Similarly, Fe_3_O_4_@Agar/HA was a viscous hydrogel, in which hyaluronic acid acted as the carrier of the Fe_3_O_4_@Agar microspheres, and Fe_3_O_4_@Agar/HA would form a mound after the injection and thus provide the bulking action as Dx/HA.

**FIGURE 2:**
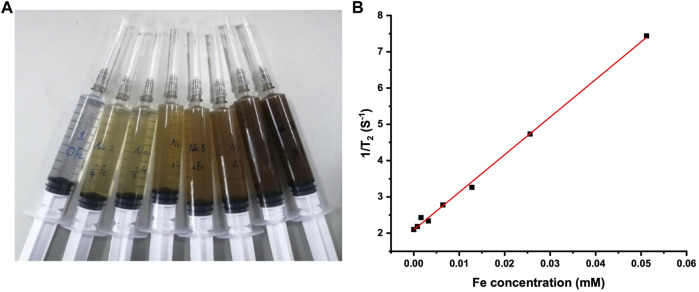
**(A)** Photo of the Fe_3_O_4_@Agar/HA samples in syringes; from left to right, the final Fe concentration was separately 0, 0.0008, 0.0016, 0.0032, 0.0064, 0.0128, 0.0256, and 0.0512 mM, the agarose and hyaluronic acid concentrations were unchanged in the all samples; **(B)** 1/T_2_ changes of the Fe_3_O_4_@Agar/HA samples versus Fe concentration.

Fe_3_O_4_ magnetic nanoparticles can be used as a T_2_-imaging contrast agent in MRI, and the T_2_-weighted contrast increases with the Fe concentration (Song et al., 2006). Figure 2A shows the photo of the Fe_3_O_4_@Agar/HA samples with the final Fe concentrations from 0 to 0.0512 mM. The transverse relaxation rate of the Fe_3_O_4_@Agar/HA samples obtained from the linear fitting of the data shown in Figure 2B was 104 mM^−1^s^−1^, which was close to the transverse relaxation rate of 123 mM^−1^s^−1^ of superparamagnetic iron Feridex, a commercial T_2_-weighted contrast agent (Xie et al., 2010). This result indicates that Fe_3_O_4_@Agar/HA can be used as a T_2_-imaging contrast agent. In the following study, the final Fe concentration in Fe_3_O_4_@Agar/HA was fixed at 0.0256 mM.

### Biological Evaluations of the Bulking Agent

#### Cytotoxicity

Table 1 shows the cell viabilities after 24 h incubation in the media containing different volume fractions of the Fe_3_O_4_@Agar/HA extract solution. The cell viability decreased with the increase of the volume fraction of the extract solution. According to GB/T 16886.5-2017/ISO 10993-5:2009 (AQSIQ and SAC, 2017a), the material has a cytotoxic potential when the cell viability is lower than 70% of the blank. In our study, the cell viability was 80% in 100% extract solution, no cell lysis was observed, and the cells were in a good growth state, indicating that Fe_3_O_4_@Agar/HA has good cytocompatibility.

**TABLE 1:** Cell viabilities after 24 h incubation in the media containing different volume fractions of Fe_3_O_4_@Agar/HA extract solution (*n* = 6).

Group	Cell morphology	Average OD_570_ ± standard deviation	Cell viability (%)
Blank	No lysis and in a good growth state	0.570 ± 0.019	100.0
100% Extract solution		0.456 ± 0.008	80.0
75% Extract solution		0.485 ± 0.022	85.1
50% Extract solution		0.493 ± 0.016	86.6
25% Extract solution		0.541 ± 0.024	95.0
Negative control		0.542 ± 0.008	95.1
Positive control	Lysis and death	0.013 ± 0.002	2.3

#### *In Vitro* Genotoxicity

The Ames test was performed to detect the point mutations (Hamada et al., 1994; Zeiger, 2019) caused by the Fe_3_O_4_@Agar/HA extract solution. For TA98, TA100, and TA102 strains, the data in Table 2 show that the revertant colony number of the extract solution group was less than two-fold of the number of the negative control group. For TA1535 and TA1537 strains, the revertant colony number of the extract solution group was less than three-fold of the number of the negative control group. These results mean that the Fe_3_O_4_@Agar/HA extract solution did not cause mutations in the stains tested (Hamada et al., 1994), indicating that Fe_3_O_4_@Agar/HA has no genotoxicity potential.

**TABLE 2:** The ratios of the revertant colony numbers of the tested groups^a^.

Bacterial strain	S9	Extract solution/negative	Positive/negative
TA98	+	1.40	71.46
	−	1.29	31.50
TA100	+	1.07	13.44
	−	0.97	11.28
TA102	+	0.90	2.64
	−	0.96	7.02
TA1535	+	0.20	8.79
	−	1.53	66.42
TA1537	+	0.28	8.34
	−	1.55	238.91

a The average revertant colony numbers and deviations of the extract solution, negative control and positive control groups are shown in Supplementary Table S1, Supplementary Table S2, and Supplementary Table S3, respectively. The negative control was saline. The positive controls are listed in Supplementary Table S3.

#### Animal Irritation

Three young adult rabbits were used for a single exposure test to assess the dermal irritation potential (AQSIQ and SAC, 2017b). Table 3 shows the erythema grade plus edema grade of each application site after the removal of Fe_3_O_4_@Agar/HA. Only one rabbit had transient minimal erythema, and no other adverse changes were observed. The primary irritation index of the Fe_3_O_4_@Agar/HA group was 0.17, calculated by dividing the sum of all the scores by 18 (three rabbits, two test sites, and three time points observed at 24, 48, and 72 h). According to GB/T 16886.10-2017/ISO 10993-10:2010 (AQSIQ and SAC, 2017b), less than 0.4 of the primary irritation index indicates that the dermal irritation potential of Fe_3_O_4_@Agar/HA is negligible.

**TABLE 3:** Erythema grade plus edema grade of each application site observed at 1, 24, 48, and 72 h after removal of Fe_3_O_4_@Agar/HA.

Group	Application site	Erythema grade plus edema grade			
		1 h	24 h	48 h	72 h
Fe_3_O_4_@Agar/HA	Rabbit 1-upper right	0	1	1	0
	Rabbit 1-lower right	0	1	0	0
	Rabbit 2-upper right	0	0	0	0
	Rabbit 2-lower right	0	0	0	0
	Rabbit 3-upper right	0	0	0	0
	Rabbit 3-lower right	0	0	0	0
Blank control	Rabbit 1-upper left	0	0	0	0
	Rabbit 1-lower left	0	0	0	0
	Rabbit 2-upper left	0	0	0	0
	Rabbit 2-lower left	0	0	0	0
	Rabbit 3-upper left	0	0	0	0
	Rabbit 3-lower left	0	0	0	0

#### Skin Sensitization

Young adult albino guinea pigs were used to assess the skin sensitization potential of the Fe_3_O_4_@Agar/HA extract solution through the guinea pig maximization test (AQSIQ and SAC, 2017b). The clinical manifestations of the guinea pigs treated with the test samples and the control samples were all normal. At 24 and 48 h after the removal of the dressings, no visible changes were observed at the challenge skin sites, suggesting that Fe_3_O_4_@Agar/HA has no skin sensitization potential.

#### Acute Systemic Toxicity

Young adult KM mice were injected with the Fe_3_O_4_@Agar/HA extract solution at a single dosage of 50 ml/kg *via* the caudal vein to assess the acute systemic toxicity. During the observation period, no mouse died, and all the mice were clinically normal. The body weight changes of the extract solution group were similar to the changes of the saline group, and the loss of the body weight was less than 5% of the corresponding weight before the injection (Table 4). After 72 h of the observations, the mice were euthanasia and dissected. No significant systemic toxicity and gross pathology changes were observed (Supplementary Figure S2). Except that some paraffin sections of the organs presented slight lymphocytic infiltration (≤20%) for both test and control groups, no significant histopathology changes were observed, as shown in Table 5. These results indicate that Fe_3_O_4_@Agar/HA has no significant acute systemic toxicity.

**TABLE 4:** Body weights of the mice before and after intravenous injection with the Fe_3_O_4_@Agar/HA extract solution or saline.

Group	Mouse No.	Body weight (g)			
		Before	After		
			1 day	2 day	3 day
Extract solution	1	33.1	32.3	33.0	32.4
	2	35.2	34.7	34.9	34.7
	3	33.0	32.8	33.7	34.3
	4	32.2	30.6	32.3	32.8
	5	36.5	34.8	36.9	36.7
Saline	1	33.9	33.9	33.2	32.9
	2	35.0	35.9	35.8	34.4
	3	36.7	37.9	37.4	35.9
	4	35.4	34.9	35.1	35.3
	5	32.7	32.9	33.2	32.6

**TABLE 5:** Histopathology observations of the paraffin sections of the murine organs excised after 72 h of the intravenous injection with Fe_3_O_4_@Agar/HA extract solution or saline.

Group	Mouse No.	Heart	Liver	Spleen	Lung	Brain
Extract solution	1	a	a	b	b	b
	2	b	b	b	a	b
	3	a	a	b	a	a
	4	a	a	a	a	a
	5	a	a	b	b	b
Saline	1	a	a	a	a	a
	2	a	b	a	b	a
	3	a	b	a	a	a
	4	a	a	a	a	a
	5	a	a	a	b	a

a: No obvious cell infiltration. b: Lymphocytic infiltration ≤20%.

### Treatment of Vesicoureteral Reflux Rabbits with the Bulking Agent

**FIGURE 3:**
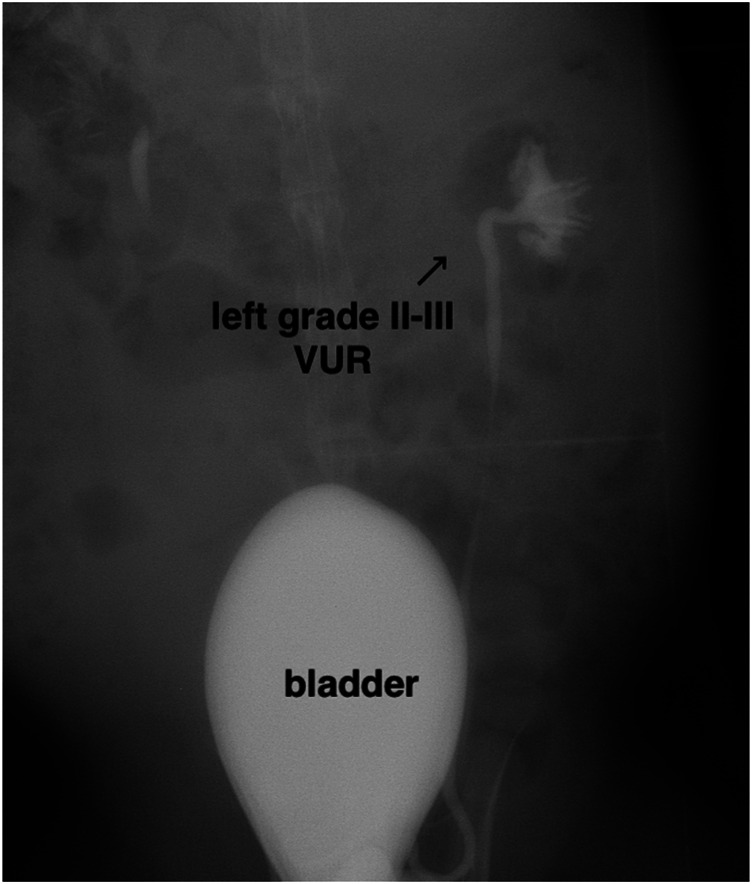
Representative VCUG image of the rabbit with II–III VUR grade in the left intravesical ureter.

**FIGURE 4:**
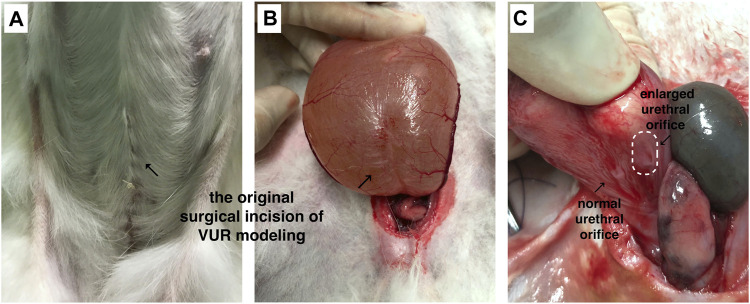
**(A,B)** The bladder was opened and the trigone of the bladder was exposed through the original surgical incisions; **(C)** the enlarged ureteral orifice is shown in the photo.

**FIGURE 5:**
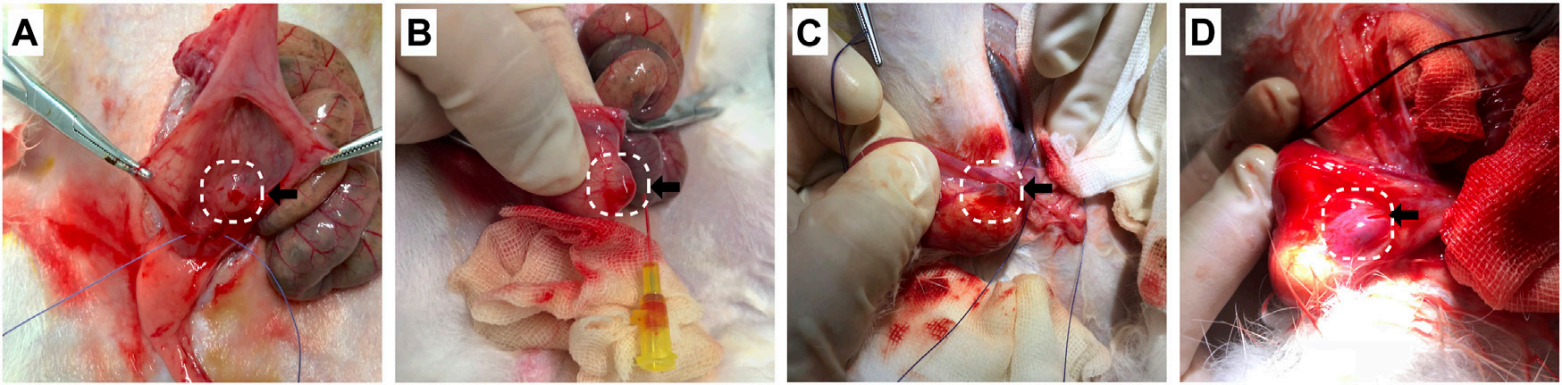
**(A−D)** The bulking agent was injected into the submucosal plane beneath the left ureteral orifice at the 6 o’clock position to create a mound to elongate the intramural ureter.

The VUR model rabbits were created by an incision of the roof of the left intravesical ureter to enlarge the ureteral orifice (Baek et al., 2010; Mangera and Edhem, 2012). The created VUR was confirmed by the VCUG examination as shown in Figure 3 after 4 weeks of the surgery. The VUR rabbits graded II−III were selected for the treatment or as the control. Because the rabbits were not big enough to use endoscope, the bulking agent or saline was injected by a subureteral transurethral injection (STING) technique through open surgery. The original surgical incisions were opened again to expose the trigone of the bladder as shown in Figure 4, in which the enlarged ureteral orifice is shown. The photos in Figure 5 show that a mound was created by the injected bulking agent that elongated the intramural ureter.

**FIGURE 6:**
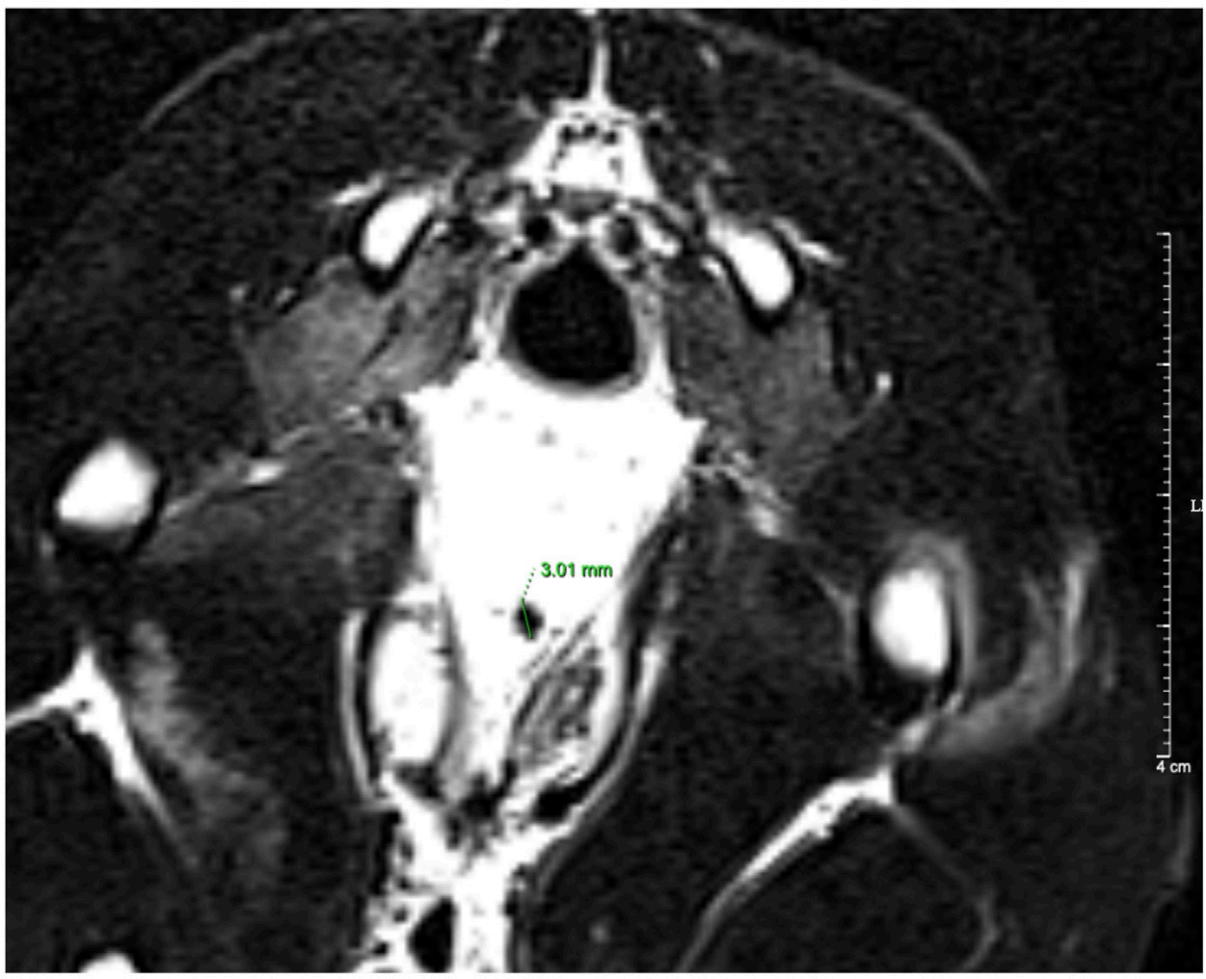
T_2_-weighted MRI image of a resolved VUR rabbit. The diameter of the bulking agent shown in the image was 3.01 mm.

Table 6 shows the treatment results of the VUR rabbits after 4 weeks of the injection. Of all 14 rabbits including 6 VUR II and 8 VUR III, 11 rabbits were alive for the analysis. In the bulking agent injection group, the VCUG examination showed that four rabbits’ VUR resolved and one rabbit’s VUR reduced from grade III to grade II. The VUR grades of the surviving rabbits in the two control groups remained unchanged. The VCUG result showed that the injection of the bulking agent Fe_3_O_4_@Agar/HA was effective in treating rabbit VUR (Fisher’s exact test *p* = 0.017). The treating efficacy was 66.7% (4/6) or 80% (4/5, excluding the unfinished rabbit). For the four resolved VUR rabbits, the bulking agent was easily detected in their bladders by T_2_-weighted MRI as shown in Figure 6. For the rabbit with a reduced VUR grade, the bulking agent was not obvious in MRI examination, suggesting that the bulking agent mound was not big enough to elongate the intramural tunnel of the ureter. The consistency of the VCUG and MRI results indicates that MRI can inspect and explain the VUR treatment effect of the injected bulking agent.

**TABLE 6:** Treatment results of the VUR rabbits after 4 weeks of the injection.

Group	Rabbit No.	Survival	VUR grade before operation	VUR grade after operation	Bulking agent on MRI-T_2_ scan
Bulking agent injection	1	Yes	II	Negative	Yes
	2	Yes	III	Negative	Yes
	3	Yes	III	Negative	Yes
	4	Died of extravasation^a^	III	—	—
	5	Yes	II	Negative	Yes
	6	Yes	III	II	Not obvious
Saline injection	1	Yes	II	II	—
	2	Yes	III	III	—
	3	Died of accidental suffocation	III	—	—
	4	Died of intestinal infection	III	—	—
Sham-operation	1	Yes	II	II	—
	2	Yes	II	II	—
	3	Yes	III	III	—
	4	Yes	II	II	—

a The VCUG image is shown in Supplementary Figure S3.

**FIGURE 7:**
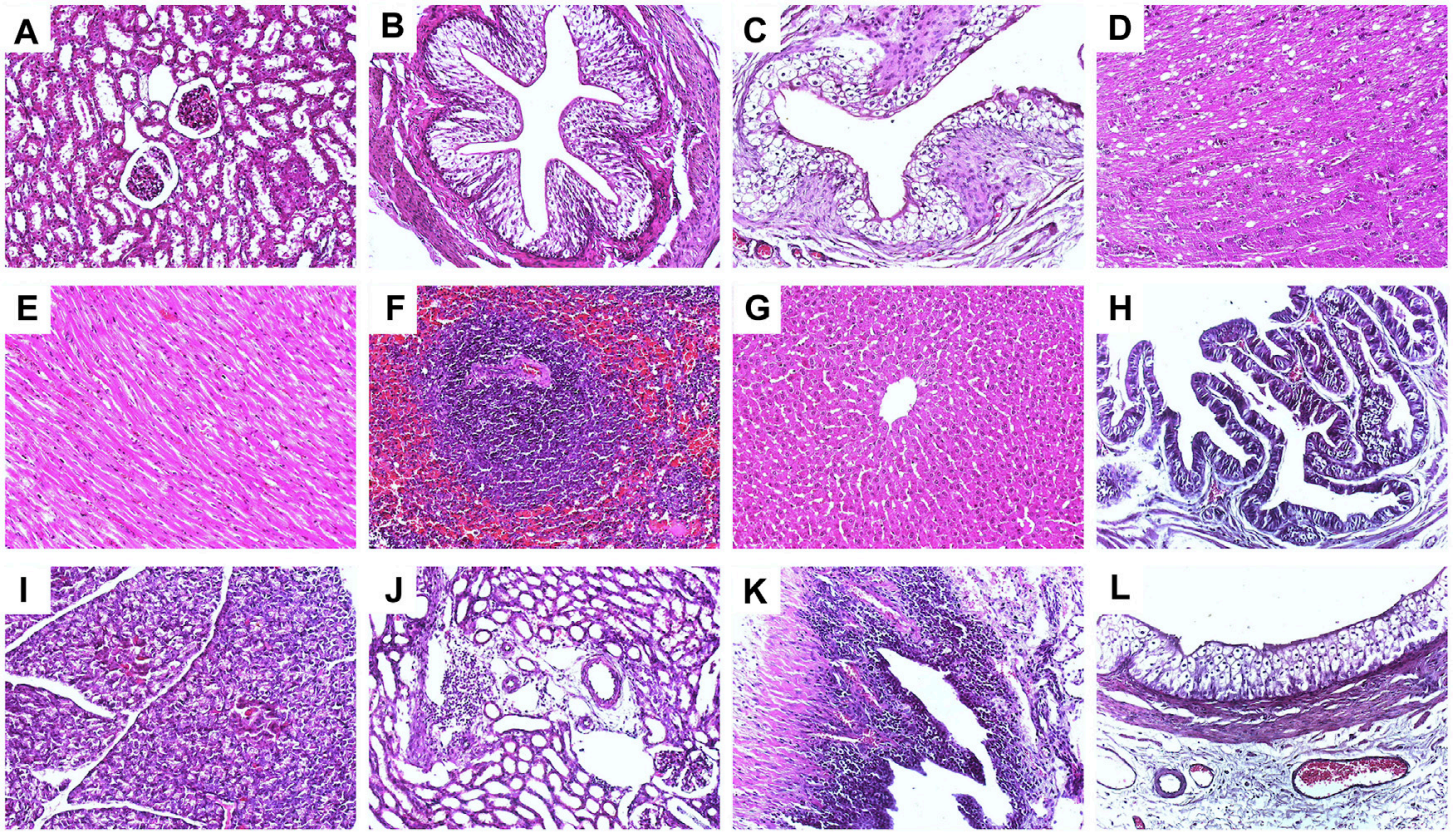
Representative hematoxylin−eosin staining histological images (×200) of the VUR rabbits in the bulking agent injection group; **(A)** right kidney, **(B)** right middle of ureter, **(C)** bladder, **(D)** brain, **(E)** heart, **(F)** spleen, **(G)** liver, **(H)** gallbladder, **(I)** pancreas, **(J)** left kidney with mild inflammatory cell infiltration in the renal interstitial, **(K)** left renal pelvis with mild mucosal edema and mild inflammatory cell infiltration, and **(L)** left ureter with mild mucosal edema.

After the VCUG and MRI examinations, all the rabbits were euthanized and dissected. The bulking agent mound was identified beneath the left ureteral orifice in each resolved VUR rabbit. The pathological analysis as shown in Figures 7A−L verified no obvious exogenous substances in the organs of all the six rabbits of the bulking agent injection group, including the rabbit with a reduced VUR grade and the unfinished rabbit, confirming that the bulking agent did not migrate during the test period. Mild inflammatory cell infiltration in the renal interstitial and renal pelvis as well as mild mucosal edema in the ureter and the renal pelvis of the left urinary system were observed in a few samples as shown in Figures 7J–L. No significant changes were observed in the other internal organs and brain, as shown in Figures 7A−I.

## Discussion

The novel magnetic bulking agent Fe_3_O_4_@Agar/HA presented good biocompatibility and biosecurity in the tests of cytotoxicity, *in vitro* genotoxicity, animal irritation, skin sensitization, and acute systemic toxicity. The pathological analysis of the rabbits showed the injected bulking agent had good *in vivo* biosecurity and biocompatibility. Agarose is mainly eliminated by the macrophages in the body (Luo and Tang, 2015), and the particles phagocytized by human macrophages are less than 80 μm (Molitierno et al., 2008). The Fe_3_O_4_@Agar microspheres were cross-linked and filtered through a sieving sieve to obtain the magnetic microspheres with diameters of 80–250 μm to avoid the dissociation, elimination, and diffusion through macrophage migration. By using a hyaluronic acid hydrogel as the carrier, the Fe_3_O_4_@Agar microspheres formed a mound after the injection into the VUR rabbit and provided an effective bulking function without migration to the organs examined. These results confirm that the Fe_3_O_4_@Agar microspheres have a long-term structural stability.

In this study, we chose a surgical method to create VUR by incising the roof of the intravesical ureter to enlarge the ureteral orifice as reported in the literature (Baek et al., 2010), which shortened the length of the intravesical ureteral tunnel and destroyed the anti-reflux mechanism of the ureterovesical junction; therefore, the resulting VUR would not resolve spontaneously over time (Mangera and Edhem, 2012). The success rate of the bulking agent in treating VUR rabbits was 67% (4/6) or 80% (4/5, excluding the unfinished rabbit), which was lower than the rate in treating VUR children (Elder et al., 2006; Chertin and Kocherov, 2010; Routh et al., 2010). The possible reasons are as follows. First, the number of the rabbits in this study was small, and the success rate was only a preliminary result. A larger number of animals will be included in the future study to obtain a reliable result of the success rate. Second, we did not have suitable endoscopy for rabbits. The bulking agent injection was performed through the open surgery, which increased the perioperative risk. Due to the damage caused by the repeated incisions of the bladder, rabbit No. 4 in the bulking agent injection group died of extravasation of urine (Supplementary Figure S3). Third, the injection technique we used is STING. It has been reported that the success rates of the hydrodistension implantation technique (HIT) and the double-HIT technique are higher than the rate of STING technique in VUR treatment. A large meta-analysis showed that the overall success rate of the HIT technique was 82.5%, while the STING technique was 71.4% (Yap et al., 2016). For the HIT, the injection is made within the ureteral orifice beneath the mucosa. The double-HIT is similar to the HIT, but two injections are performed (Blais et al., 2017). Because the rabbits have a very thin ureter and bladder wall, it is very difficult for HIT treatment. Therefore, the STING technique was chosen in this study. In the future study, we plan to choose larger animals such as pigs or dogs, and use the endoscopic HIT technique to completely simulate human VUR treatment.

MRI is a noninvasive method and commonly used in clinical examination. As shown in Figure 6, the bulking agent in the bladder was visible in the magnetic resonance image after 4 weeks of the injection, demonstrating that the bulking agent is trackable through MRI. The cross-linked Fe_3_O_4_@Agar microspheres protected the Fe_3_O_4_ nanoparticles from decomposition and migration that enable the bulking agent to have a long-term trackable property. This property is very useful for clinicians to make decisions when the complications such as persistent VUR and ureteral obstruction occur after the endoscopic injection.

## Conclusion

A novel magnetic bulking agent, namely, Fe_3_O_4_@Agar/HA, was produced for endoscopic VUR treatment as well as for the inspection of the treatment effect. The bulking agent was produced by embedding Fe_3_O_4_ magnetic nanoparticles in cross-linked agarose microspheres Fe_3_O_4_@Agar and dispersing Fe_3_O_4_@Agar in a hyaluronic acid hydrogel. The bulking agent has good biocompatibility and biosecurity proved by the tests of cytotoxicity, *in vitro* genotoxicity, animal irritation, skin sensitization, acute systemic toxicity, and pathological analysis. The success rate of the bulking agent in treating VUR rabbits by injection was 80%, and no migrated particles were found in the organs of the rabbits. After injection, the bulking agent was long-term trackable through MRI that can help clinicians to inspect the VUR treatment effect. This study shows that the bulking agent with a long-term stable tracer is promising for endoscopic VUR treatment.

## Data Availability

The original contributions presented in the study are included in the article/Supplementary Material; further inquiries can be directed to the corresponding authors.

## References

[B1] Alizadeh F. Omidi I. Haghdani S. Hatef Khorrami M. Izadpanahi M. H. Mohammadi Sichani M. (2019). A Comparison Between Dextranomer/ Hyaluronic Acid and Polyacrylate Polyalcohol Copolymer as Bulking Agents for Treating Primary Vesicoureteral Reflux. Urol. J. 16, 174–179. 10.22037/uj.v0i0.4156 30178456

[B2] AQSIQ and SAC (2010). Laboratory Animal - Requirements of Environment and Housing Facilities (GB14925-2010). Beijing: Standards Press of China.

[B3] AQSIQ and SAC (2011a). Chinese National Standard of Biological Evaluation of Medical Devices - Part 1: Evaluation and Testing in the Risk Management Process (GB/T 16886.1-2011/ISO 10993-1:2009). Beijing: Standards Press of China.

[B4] AQSIQ and SAC (2011b). Chinese National Standard of Biological Evaluation of Medical Devices - Part 11: Tests for Systemic Toxicity (GB/T 16886.11-2011/ISO 10993-11:2006). Beijing: Standards Press of China.

[B5] AQSIQ and SAC (2017a). Chinese National Standard of Biological Evaluation of Medical Devices - Part 5: Test for *in Vitro* Cytotoxicity (GB/T 16886.5-2017/ISO 10993-5:2009). Beijing: Standards Press of China.

[B6] AQSIQ and SAC (2017b). Chinese National Standard of Biological Evaluation of Medical Devices - Part 10: Tests for Irritation and Skin Sensitization (GB/T 16886.10-2017/ISO 10993-10:2010). Beijing: Standards Press of China.

[B7] Arlen A. M. Cooper C. S. (2015). Controversies in the Management of Vesicoureteral Reflux. Curr. Urol. Rep. 16, 64. 10.1007/s11934-015-0538-2 26199037

[B8] Baek M. Paick S. H. Jeong S. J. Hong S. K. Kim S. W. Choi H. (2010). Urodynamic and Histological Changes in a Sterile Rabbit Vesicoureteral Reflux Model. J. Korean Med. Sci. 25, 1352–1358. 10.3346/jkms.2010.25.9.1352 20808680 PMC2923783

[B9] Blais A.-S. Bolduc S. Moore K. (2017). Vesicoureteral Reflux: From Prophylaxis to Surgery. Can. Urol. Assoc. J. 11, 13–S18. 10.5489/cuaj.4342 28265309 PMC5332225

[B10] Cerwinka W. H. Scherz H. C. Kirsch A. J. (2008). Endoscopic Treatment of Vesicoureteral Reflux With Dextranomer/Hyaluronic Acid in Children. Adv. Urol. 2008, 1–7. 10.1155/2008/513854 PMC2441859 18604293

[B11] Ceylan T. Çıtamak B. Bozacı A. C. Altan M. Gasimov K. Asi T. (2021). Endoscopic Treatment of Vesicoureteral Reflux: Changing Trends Over the Years. J. Urol. Surg. 8, 123–129. 10.4274/jus.galenos.2021.0001

[B12] Chertin B. Kocherov S. (2010). Long-Term Results of Endoscopic Treatment of Vesicoureteric Reflux With Different Tissue-Augmenting Substances. J. Pediatr. Urol. 6, 251–256. 10.1016/j.jpurol.2009.10.011 19896419

[B13] Diamond D. A. Mattoo T. K. (2012). Endoscopic Treatment of Primary Vesicoureteral Reflux. N. Engl. J. Med. 366, 1218–1226. 10.1056/NEJMct1108922 22455416

[B14] Elder J. S. Diaz M. Caldamone A. A. Cendron M. Greenfield S. Hurwitz R. (2006). Endoscopic Therapy for Vesicoureteral Reflux: a Meta-Analysis. I. Reflux Resolution and Urinary Tract Infection. J. Urol. 175, 716–722. 10.1016/S0022-5347(05)00210-7 16407037

[B15] Elmore J. M. Kirsch A. J. Heiss E. A. Gilchrist A. Scherz H. C. (2008). Incidence of Urinary Tract Infections in Children After Successful Ureteral Reimplantation Versus Endoscopic Dextranomer/Hyaluronic Acid Implantation. J. Urol. 179, 2364–2368. 10.1016/j.juro.2008.01.149 18436248

[B16] Hamada C. Wada T. Sakamoto Y. (1994). Statistical Characterization of Negative Control Data in the Ames Salmonella/Microsome Test. Environ. Health Perspect. 102 (Suppl. 1), 115–119. 10.1289/ehp.94102s1115 PMC1566914 8187699

[B17] Herbst K. W. Corbett S. T. Lendvay T. S. Caldamone A. A. (2014). Recent Trends in the Surgical Management of Primary Vesicoureteral Reflux in the Era of Dextranomer/Hyaluronic Acid. J. Urol. 191, 1628–1633. 10.1016/j.juro.2013.09.055 24679885

[B18] Johnston D. L. Qureshi A. H. Irvine R. W. Giel D. W. Hains D. S. (2016). Contemporary Management of Vesicoureteral Reflux. Curr. Treat. Options Peds. 2, 82–93. 10.1007/s40746-016-0045-9 PMC4996282 27570729

[B19] Kershen R. T. Atala A. (1999). New Advances in Injectable Therapies for the Treatment of Incontinence and Vesicoureteral Reflux. Urol. Clin. North America 26, 81–94. 10.1016/s0094-0143(99)80008-1 10086052

[B20] Keshel S. H. Rahimi A. Hancox Z. Ebrahimi M. Khojasteh A. Sefat F. (2020). The Promise of Regenerative Medicine in the Treatment of Urogenital Disorders. J. Biomed. Mater. Res. 108, 1747–1759. 10.1002/jbm.a.36942 32270582

[B21] Kim S. W. Lee Y. S. Han S. W. (2017). Endoscopic Injection Therapy. Investig. Clin. Urol. 58, S38–S45. 10.4111/icu.2017.58.S1.S38 PMC5468263 28612059

[B22] Kim S. W. Lee Y. S. Im Y. J. Han S. W. (2018). New Bulking Agent for the Treatment of Vesicoureteral Reflux: Polymethylmethacrylate/Dextranomer. Investig. Clin. Urol. 59, 206–212. 10.4111/icu.2018.59.3.206 PMC5934284 29744479

[B23] Kirsch A. Hensle T. Scherz H. Koyle M. (2006). Injection Therapy: Advancing the Treatment of Vesicoureteral Reflux. J. Pediatr. Urol. 2, 539–544. 10.1016/j.jpurol.2005.12.004 18947677

[B24] Kirsch A. J. Arlen A. M. (2014). Evaluation of New Deflux Administration Techniques: Intraureteric HIT and Double HIT for the Endoscopic Correction of Vesicoureteral Reflux. Expert Rev. Med. Devices 11, 439–446. 10.1586/17434440.2014.929491 24931132

[B25] Laurent S. Forge D. Port M. Roch A. Robic C. Vander Elst L. (2008). Magnetic Iron Oxide Nanoparticles: Synthesis, Stabilization, Vectorization, Physicochemical Characterizations, and Biological Applications. Chem. Rev. 108, 2064–2110. 10.1021/cr068445e 18543879

[B26] Lee E. K. Gatti J. M. Demarco R. T. Murphy J. P. (2009). Long-Term Followup of Dextranomer/Hyaluronic Acid Injection for Vesicoureteral Reflux: Late Failure Warrants Continued Followup. J. Urol. 181, 1869–1875. 10.1016/j.juro.2008.12.005 19233403

[B27] Luo S. S. Tang S. Q. (2015). Research Progress of Agarose in Tissue Engineering. China Biotechnol. 35, 68–74. 10.13523/j.cb.2015061

[B28] Mangera A. Edhem I. (2012). Bladder Wrap: a Technique to Restore Continence in an Incompetent Vesicocutaneous Diversion. Ann. R. Coll. Surg. Engl. 94, 442–443. 10.1308/003588412X13373405386015j PMC3954336 22943345

[B29] Molitierno J. A. Scherz H. C. Kirsch A. J. (2008). Endoscopic Treatment of Vesicoureteral Reflux Using Dextranomer Hyaluronic Acid Copolymer. J. Pediatr. Urol. 4, 221–228. 10.1016/j.jpurol.2007.11.015 18631931

[B30] Montini G. Tullus K. Hewitt I. (2011). Febrile Urinary Tract Infections in Children. N. Engl. J. Med. 365, 239–250. 10.1056/NEJMra1007755 21774712

[B31] Moore K. Bolduc S. (2014). Prospective Study of Polydimethylsiloxane vs Dextranomer/Hyaluronic Acid Injection for Treatment of Vesicoureteral Reflux. J. Urol. 192, 1794–1800. 10.1016/j.juro.2014.05.116 24928269

[B32] O'donnell B. Puri P. (1984). Treatment of Vesicoureteric Reflux by Endoscopic Injection of Teflon. Bmj. 289, 7–9. 10.1136/bmj.289.6436.7 6428669 PMC1442065

[B33] Park S. H. Lee C.-R. Hong S.-K. (2020). Implications of Agar and Agarase in Industrial Applications of Sustainable Marine Biomass. Appl. Microbiol. Biotechnol. 104, 2815–2832. 10.1007/s00253-020-10412-6 32036436

[B34] Peters C. A. Skoog S. J. Arant B. S. Jr. Copp H. L. Elder J. S. Hudson R. G. (2010). Summary of the AUA Guideline on Management of Primary Vesicoureteral Reflux in Children. J. Urol. 184, 1134–1144. 10.1016/j.juro.2010.05.065 20650499

[B35] Renkema K. Y. Winyard P. J. Skovorodkin I. N. Levtchenko E. Hindryckx A. Jeanpierre C. (2011). Novel Perspectives for Investigating Congenital Anomalies of the Kidney and Urinary Tract (CAKUT). Nephrol. Dial. Transplant. 26, 3843–3851. 10.1093/ndt/gfr655 22121240

[B36] Routh J. C. Inman B. A. Reinberg Y. (2010). Dextranomer/Hyaluronic Acid for Pediatric Vesicoureteral Reflux: Systematic Review. Pediatrics 125, 1010–1019. 10.1542/peds.2009-2225 20368325

[B37] Şencan A. Vatansever S. Yilmaz Ö. Genç A. Serter S. Gümüşer G. (2008). Early Renal Parenchymal Histological Changes in an Experimental Model of Vesico-Ureteral Reflux and the Role of Apoptosis. Scand. J. Urol. Nephrol. 42, 213–219. 10.1080/00365590701701632 17943638

[B38] Simoes E. Silva A. C. Silva J. M. P. Diniz J. S. S. Pinheiro S. V. B. Lima E. M. (2007). Risk of Hypertension in Primary Vesicoureteral Reflux. Pediatr. Nephrol. 22, 459–462. 10.1007/s00467-006-0349-2 17143629

[B39] Sizonov V. V. Kagantsov I. M. Mayr J. M. Akramov N. R. Pirogov A. V. Gasanov Z. A. (2020). Risk Factors for Obstructive Complications after Endoscopic Correction of Vesico-Ureteral Reflux Using Polyacrylate Polyalcohol Copolymer. Medicine (Baltimore). 99, e20386. 10.1097/MD.0000000000020386 32481425 PMC12245303

[B40] Skoog S. J. Peters C. A. Arant B. S. Jr. Copp H. L. Elder J. S. Hudson R. G. (2010). Pediatric Vesicoureteral Reflux Guidelines Panel Summary Report: Clinical Practice Guidelines for Screening Siblings of Children With Vesicoureteral Reflux and Neonates/Infants With Prenatal Hydronephrosis. J. Urol. 184, 1145–1151. 10.1016/j.juro.2010.05.066 20650494

[B41] Song R. Lin W. Wehrli F. W. Chen Q. Asakura T. Song H. (2006). Transverse Relaxation Rates (R2*, R2, and R2') of Iron-Overloaded Livers of Thalassemic Mice at 1.5T and 3T. Proc. Int. Soc. Magn. Reson. Med. 14, 3305.

[B42] Xie J. Chen K. Huang J. Lee S. Wang J. Gao J. (2010). PET/NIRF/MRI Triple Functional Iron Oxide Nanoparticles. Biomaterials 31, 3016–3022. 10.1016/j.biomaterials.2010.01.010 20092887 PMC2838491

[B43] Xu Z. Xia A. Wang C. Yang W. Fu S. (2007). Synthesis of Raspberry-Like Magnetic Polystyrene Microspheres. Mater. Chem. Phys. 103, 494–499. 10.1016/j.matchemphys.2007.02.074

[B44] Xu Z. Z. Wang C. C. Yang W. L. Deng Y. H. Fu S. K. (2004). Encapsulation of Nanosized Magnetic Iron Oxide by Polyacrylamide via Inverse Miniemulsion Polymerization. J. Magnetism Magn. Mater. 277, 136–143. 10.1016/j.jmmm.2003.10.018

[B45] Yap T.-L. Chen Y. Nah S. A. Ong C. C. P. Jacobsen A. Low Y. (2016). STING Versus HIT Technique of Endoscopic Treatment for Vesicoureteral Reflux: A Systematic Review and Meta-Analysis. J. Pediatr. Surg. 51, 2015–2020. 10.1016/j.jpedsurg.2016.09.028 27773360

[B46] Zeiger E. (2019). The Test That Changed the World: The Ames Test and the Regulation of Chemicals. Mutat. Research/Genetic Toxicol. Environ. Mutagenesis 841, 43–48. 10.1016/j.mrgentox.2019.05.007 31138410

