# Supplementary material for: Magnetic Agarose Microspheres/Hyaluronic Acid Hydrogel as a Trackable Bulking Agent for Vesicoureteral Reflux Treatment
Source: Front Bioeng Biotechnol. 2021 Oct 7;9:746609. doi: 10.3389/fbioe.2021.746609 (PMC8529187; doi:10.3389/fbioe.2021.746609)
Supplement: Supplementary file 1 [file Table1.docx]

**Supplementary Material**

**Table S1.** Revertant colony numbers of the Fe_3_O_4_@Agar@HA extract solution groups (n = 3).

| **Strain** | **S9** | **Number** |
| --- | --- | --- |
| TA98 | + | 16.33 ± 8.39 |
|  | − | 12.00 ± 2.65 |
| TA100 | + | 104.67 ± 4.16 |
|  | − | 75.33 ±2.52 |
| TA102 | + | 433.33 ± 149.26 |
|  | − | 230.67 ± 90.89 |
| TA1535 | + | 7.67 ± 2.52 |
|  | − | 9.67 ± 0.58 |
| TA1537 | + | 5.00 ± 1.00 |
|  | − | 5.67 ± 0.58 |

**Table S2.** Revertant colony numbers of the negative control (saline) groups (n = 3).

| **Strain** | **S9** | **Number** |
| --- | --- | --- |
| TA98 | + | 11.67 ± 1.53 |
|  | − | 9.33 ± 4.16 |
| TA100 | + | 97.67 ± 8.62 |
|  | − | 77.67 ± 11.06 |
| TA102 | + | 484.00 ± 46.13 |
|  | − | 240.00 ± 18.33 |
| TA1535 | + | 38.67 ± 3.21 |
|  | − | 6.33 ± 2.08 |
| TA1537 | + | 17.67 ± 6.43 |
|  | − | 3.67 ± 0.58 |

**Table S3.** Revertant colony numbers of the positive control groups (n = 3).

| **Strain** | **Mutagenic agent** | **S9** | **Number** |
| --- | --- | --- | --- |
| TA98 | benzopyrene | + | 833.67 ± 175.56 |
|  | 2-nitro fluorene | − | 294.00 ± 91.94 |
| TA100 | 2-aminoanthracene | + | 1313.00 ± 91.70 |
|  | sodium azide | − | 876.00 ± 56.71 |
| TA102 | 2-aminoanthracene | + | 1276.00 ± 161.34 |
|  | methylmesylate | − | 1685.33 ± 124.02 |
| TA1535 | 2-aminoanthracene | + | 340.00 ± 20.00 |
|  | sodium azide | − | 420.67 ± 48.43 |
| TA1537 | 2-aminoanthracene | + | 147.33 ± 86.03 |
|  | ICR191 | − | 876.00 ± 135.23 |


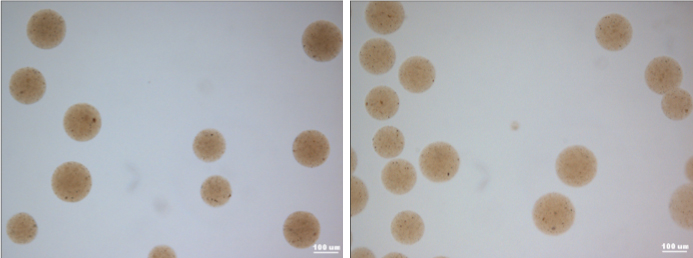


**Figure S1.** Optical microscopy images of the Fe_3_O_4_@Agar microspheres before (left) and after (right) sterilization by autoclaving.


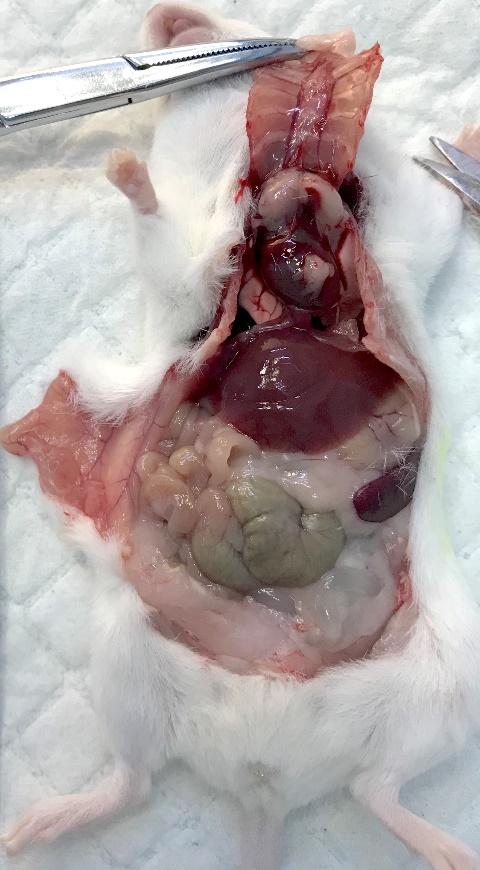


**Figure S2.** Representative gross pathology observation of the mouse after 72 h of the intravenous injection with Fe_3_O_4_@Agar/HA extract solution.

***
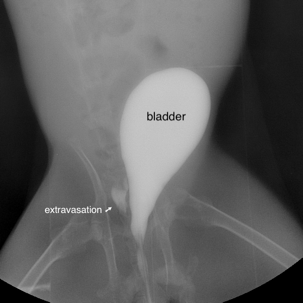
***

**Figure S3.** VCUG image of the rabbit No. 4 in the bulking agent injection group. The rabbit died of the extravasation of urine.
